# FGF21 protects against ox-LDL induced apoptosis through suppressing CHOP expression in THP1 macrophage derived foam cells

**DOI:** 10.1186/s12872-015-0077-2

**Published:** 2015-07-30

**Authors:** En Li, Ting Wang, Feng Wang, Tao Wang, Li-qiang Sun, Li Li, Shao-hui Niu, Jin-ying Zhang

**Affiliations:** Department of Cardiovascular internal medicine, The first Affiliated Hospital of Zhengzhou University, Zhengzhou, P. R. China; Department of Cardiovascular internal medicine, The second Affiliated Hospital of Zhengzhou University, Zhengzhou, P. R. China; Department of Gerontology, Shaanxi People’s Hospital, Xi’an, P. R. China; CAS Key Laboratory of Computational Biology, CAS-MPG Partner Institute for Computational Biology, Shanghai Institutes for Biological Sciences, Chinese Academy of Sciences, Shanghai, P. R. China; Department of Gerontology, The second Affiliated Hospital of Zhengzhou University, Zhengzhou, P. R. China

**Keywords:** Macrophage, ER stress, FGF21, Foam cell, CHOP

## Abstract

**Background:**

FGF21,as a member of the fibroblast growth factor superfamily, is an important endogenous regulator to systemic glucose and lipid metabolism. Elevated serum FGF21 levels have been reported in subjects with coronary heart disease and carotid artery plaques. The formation and apoptosis of foam cell, induced by ox-LDL and oxysterols, are key steps in the development of atherosclerosis.

**Methods:**

In this study, THP1 derived macrophages were induced into foam cells by ox-LDL or sterols. The formation and apoptosis of foam cells treated with or without FGF21 were analyzed.

**Results:**

We demonstrated that the accumulation of cholesterol was decreased after FGF21 treatment in THP1 macrophage derived foam cells. Consistently, the apoptosis of macrophage was alleviated dramatically with FGF21 treatment. ERK1/2 knockdown didn’t abrogate the effect of FGF21 on THP1 macrophage derived foam cells. However, FGF21 suppressed the induced expression of CHOP and DR5 in THP1 macrophage derived foam cells.

**Conclusion:**

FGF21 protects against the formation and apoptosis of THP1 macrophages derived foam cells through suppressing the expression of CHOP.

## Background

Fibroblast growth factor (FGF) superfamily,, has an important functions in metabolic processes. Among members of the FGF superfamily, FGF21 plays a very important regulatory role in glucose and lipid homeostasis [1–3].

The role of FGF21 in glucose and lipid metabolism has been well studied [4, 5]. Human recombinant FGF21 has been demonstrated to stimulate glucose incorporation in mouse and human adipocytes, and to lower blood glucose and triglyceride levels in diabetic and obese mice as well as diabetic monkeys [6]. By contrast, FGF21 deficient mice showed mild weight gain, slightly impaired glucose homeostasis, and also developed hepatosteatosis and obvious impairments in ketogenesis and glucose control when raised on a ketogenic diet [7]. These findings suggest that FGF21 is an important metabolic hormone in maintaining glucose and lipid homeostasis. Recently, it is reported that serum FGF21 levels are increased in coronary heart disease (CHD) and FGF21 is also found in carotid artery plaques [5, 8, 9]. In myocardial infarction, FGF21could attenuate pathological myocardial remodeling through the adiponectin-dependent mechanism [10]. What’s more, FGF21 have also been found to attenuate hyperlipidemia and diabetes induced early-stage apoptosis [11]. Based on these results, FGF21 has been proposed to be associated with arteriosclerosis. However, the role of FGF21 in arteriosclerosis remains unclear.

In normal macrophages, low density lipoprotein (LDL) cholesterol particles are loaded from late endosomes to the ER. In the ER, cholesterol is esterified and accumulated to form inert lipid droplets [12, 13]. In atherosclerotic macrophages, ER-mediated cholesterol reesterification is markedly impaired resulting in excessive intracellular deposits of nonesterified cholesterol and the formaition of foam cells [13], where intraluminal ER oxidoreductases oxidize cholesterol to 7-ketocholesterol (7-KC) and other oxysterols. Oxysterols are highly cytotoxic and may induce cell death through ROS-mediated oxidative damage [14]. Prolonged ER stress contributes to apoptosis of lesional macrophages, which is associated with robust expression of C/EBP homologous protein (CHOP) in human lesions [15] and atherosclerotic plaques of apolipoprotein (apo) E-deficient mice [16]. Inactivating CHOP in apoE-deficient mice slows down macrophage apoptosis and plaque necrosis [17–19]. CHOP contributes to ER stress-induced macrophage death by inducing Fas activation, depletion of ER-associated calcium stores, and release of apoptogens from mitochondria [19]. Moreover, CHOP is found to induce cell apoptosis through activating death receptor 5 (DR5) in human carcinoma cells [20].

## Methods

### Isolation and oxidation of Low density lipoprotein

The native low-density lipoprotein (LDL) was obtained from Sigma. LDL was oxidized with CuSO_4_ at 37 °C for 18 h and transferred into ethylene diamine tetraacetic acid (EDTA; 200 mM) in phosphate-buffered saline (PBS) for 24 h at 4 °C to remove Cu^2+^. Subsequently, the product was dialyzed in PBS for 24 h at 4 °C to remove EDTA. LDL oxidation was confirmed by thiobarbituric acid reaction substances with malondialdehyde as the standard. The content of ox-LDL was 1.12 compared with 0.30 nmol/100 mg protein in the native LDL preparation (p < 0.01). The ox-LDL was then sterilized by filtration and stored at 4 °C as previously described [21].

### Cell Culture

The human THP-1 cells were obtained from the Type Culture Collection of the Chinese Academy of Sciences (Shanghai, China). THP-1 cells were cultured in Roswell Park Memorial Institute medium 1640 (RPMI 1640, Hyclone) containing 10 % fetal bovine serum and 2 mM L-glutamine. The cells were differentiated into macrophages by adding 100 ng/mL phorbol 12-myristate-13-acetate for 24 h, and the medium was then replaced with that containing ox-LDL (50 mg/mL) and human FGF21 (Peprotech, 20 nmol/L) for 24 h to obtain fully differentiated foam cells before use in experiments. And human FGF21 (Peprotech, 20 nmol/L) was added to cotreat the THP1 macrophage derived foam cell.

### SiRNA transfection

THP-1 cells were transfected with specific siRNA oligomers directed against Erk (80 nM) using Lipofectaminqe 2000 transfection reagent (Invitrogen, Carlsbad, CA) according to the manufacturer’s instructions. Negative control siRNA oligomers were used as a negative control. After transfection for 48 h, the cells were exposed to ox-LDL (50 mg/L) for 24 h. The silencing of target genes was validated by western blotting.

### Western blotting

Total proteins and nuclear proteins from cells were extracted using RIPA lysis buffer and nuclear extraction kits, respectively, following the manufacturer’s instructions. Equal amounts of protein were separated by SDS-PAGE and probed with various primary antibodies as indicated. Immunoblots were visualized using ECL reagent, and the integrated optical density (IOD) of immunoreactive bands was measured using Image-Pro Plus software and normalized by house-keeping protein (GAPDH).

### Quantitative real-time PCR

Total RNA was extracted using Trizol reagent (Invitrogen). 2 μg of total RNA was reversely transcripted using MMLV Reverse Transcriptase (Invitrogen). Real-time PCR was performed on a Rotor-Gene Q real-time PCR cycler (Roche, Shanghai, China) using SYBR-green PCR master mix kits. The data were analyzed using the Rotor-Gene Q software (version 1.7, Qiagen), and relative mRNA levels were calculated using the 2^--△△Ct^ method and normalized against 18S rRNA. The primers used for real-time PCR were synthesized by Sangon Biotech (Shanghai, China).

### Statistical analyses

Analysis of variance was conducted to examine whether significant (*P*, 0.05) main treatment and time effects occurred. Additional post hoc comparisons of treatment means were conducted by using the Dunnett’s *t*-test (treatments vs. controls) and Bonferroni *t*-test (selected comparisons) as indicated. Data given represent meansstandard deviation.

## Results

### FGF21 decreases the formation of foam cell fromTHP1 derived macrophages

**Fig. 1:**
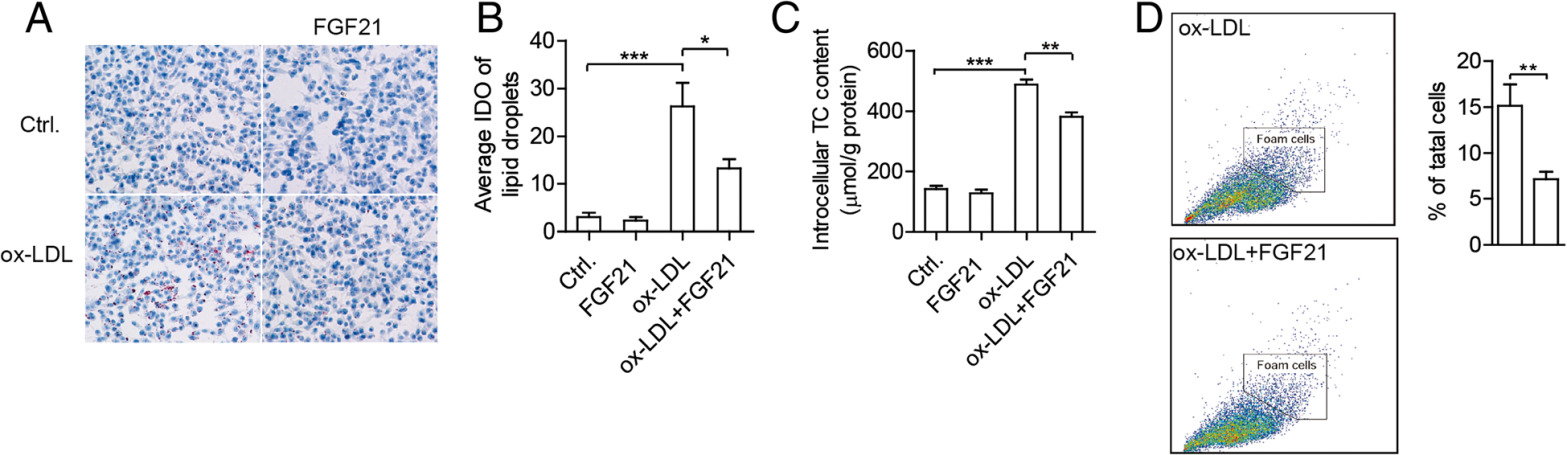
FGF21 decreased foam cell formation of THP1 derived macrophages. **a** Representative images and (**b**) the average integrated optical density (*IOD*) of lipid droplets stained with oil red O from differentiated macrophages treated by 50 mg/L ox-LDL in the presence or absence of 20 nmol/L FGF21 for 24 h. **c** The intracellular total cholesterol (TC) content was measured under the same conditions as in **a**. **d** Foam cells were identified by FACS assay, representative images of FACS (left) and the percentages of apoptosis cells (right). Data are presented as the mean ± S.E. of at least four independent experiments. *, *p <* 0.05, **, *p* < 0.01

With the development of atherosclerosis, the infiltrated macrophages engulf LDL leading to the accumulation of cholesterol in the droplets, and the formation of foam cells. The uptake of ox-LDL by macrophages is the event that triggers the formation of lipid-laden foam cells. The oil red O-staining (Fig. 1a and b) and intracellular TC quantitative assay (Fig. 1c) indicated that lipid content in differentiated THP-1 cells was significantly increased by ox-LDL, indicating the formation of foam cells. When the cells were co-treated with FGF21, the lipid content decreased dramatically as shown by the reduced lipid droplets (Fig. 1a and b) and lower intracellular TC (Fig. 1c). FACS analysis also demonstrated that ox-LDL induced foam cells decreased dramatically after FGF21 treatment (Fig. 1d). These results show that FGF21 can decrease the accumulation of cholesterol and inhibit foam cell formation in macrophages treated by ox-LDL.

### FGF21 protects against ox-LDL and 7-KC induced macrophage apoptosis

**Fig. 2:**
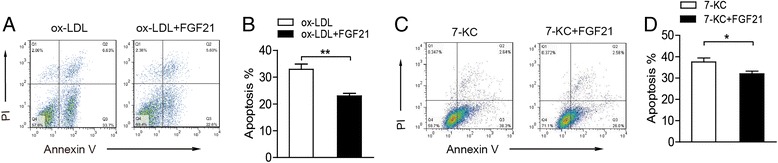
FGF21 protected against ox-LDL and 7-KC induced macrophage apoptosis. **a** THP1 derived macrophages apoptosis was test by Annexin V and PI staining treated by ox-LDL in the presence or absence of FGF21 for 24 h. **b** Quantification of apoptosis cells in the FACS assay results of **a**. **c** THP1 derived macrophages apoptosis was test by Annexin V and PI staining treated by 7-KC in the presence or absence of FGF21 for 24 h. **d** Quantification of apoptosis cells in the FACS assay results of **c**. Data are presented as the mean ± S.E. of at least four independent experiments. *, *p <* 0.05, **, *p* < 0.01

The apoptosis of foam cells duo to over-loaded cholesterol is the key event during the development of atherosclerosis,. We wondered about the effect of FGF21 on the apoptosis of macrophages induced by ox-LDL. As demonstrated by FACS analysis results, the apoptosis of THP1 derived macrophages increased significantly after the treatment of ox-LDL (Fig. 2a and b). Consist with the inhibitory effect of FGF21 on foam cell formation, the apoptosis of THP1 derived macrophages induced by the uptake of ox-LDL decreased dramatically when the cells were treated by FGF21 (Fig. 2a and b). Ox-LDL is composed of many potentially proatherogenic molecules. 7-ketocholesterol (7-KC), as one of the oxysterols, is found in relatively large abundance in ox-LDL, as well as in atherosclerotic plaque and foam cells in vivo. We wondered if FGF21 had the same effect on 7-KC induced apoptosis of macrophages. As the results of FACS analysis, FGF21 inhibited significantly the 7-KC induced apoptosis of THP1 derived macrophages (Fig. 2c and d). These results revealed that FGF21 protected against ox-LDL and oxysterols induced macrophage apoptosis.

### The effect of FGF21 on macrophages is independent upon ERK signaling pathway

**Fig. 3:**
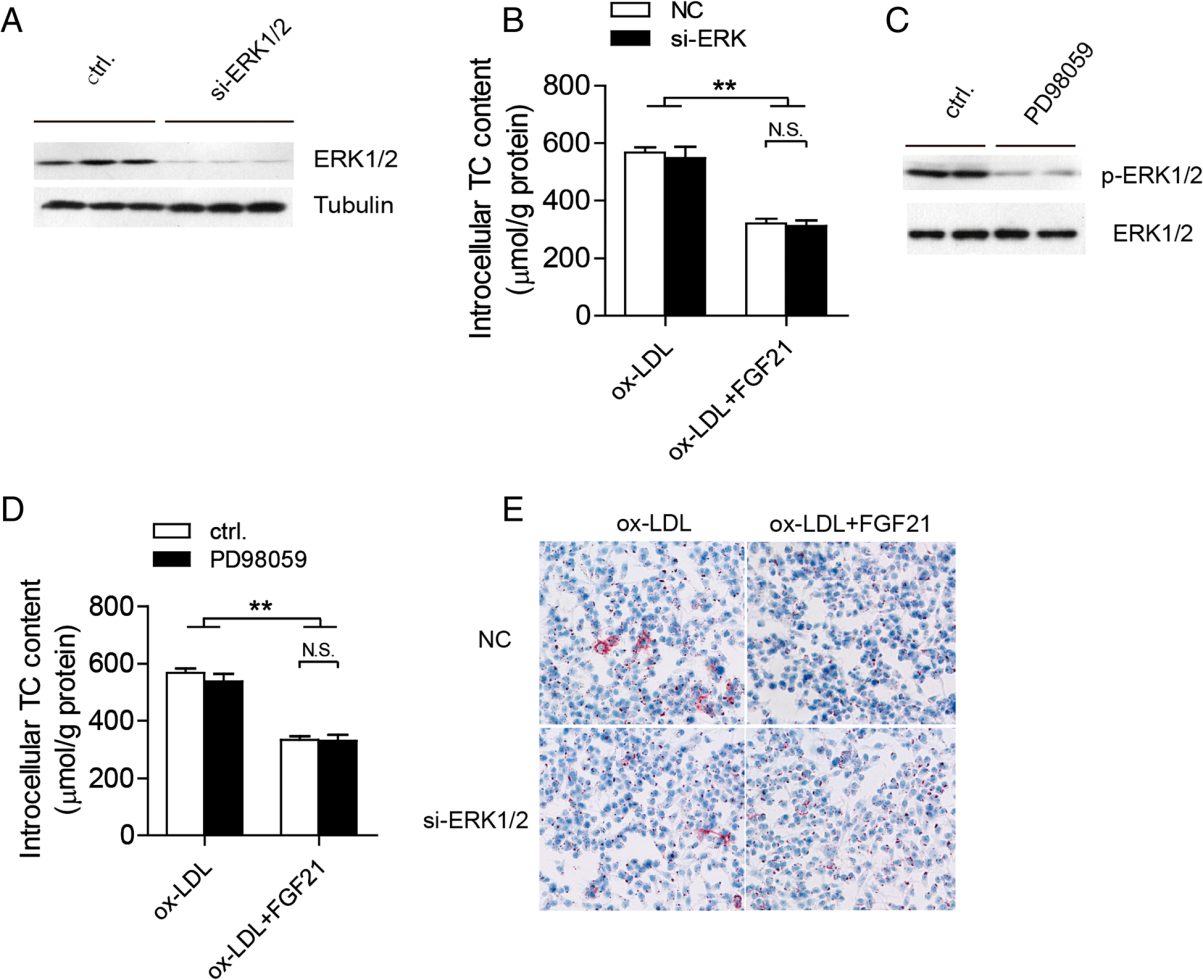
The effect of FGF21 on macrophages is ERK signaling pathway independent. **a** The knockdown efficiency of ERK1/2 was tested by western blotting in the THP1 derived macrophages transfected by siRNA. **c** Phosphorylated ERK1/2 level was tested in THP1 derived macrophages with or without PD98059 treatment for 24 h. The intracellular total cholesterol (TC) content was measured in the THP1 derived macrophages treated by ox-LDL in the presence or absence of FGF21 with ERK1/2 knockdown **b** or ERK1/2 specific inhibitor PD98059 **d**. Data are presented as the mean ± S. **e**. of at least four independent experiments. *, *p <* 0.05, **, *p* < 0.01. N.S, no significance

ERK signaling pathway plays significant role in FGF21 function [20, 22]. To test if the inhibition function of FGF21 on foam cell formation and apoptosis of macrophages is ERK pathway dependent, siRNA selectively targeted ERK1/2 were used to knocked down the protein in macrophages. Western blotting results showed that ERK1/2 protein levels decreased dramatically after targeted siRNA transfection in macrophages (Fig. 3a). As shown in Fig. 3b, in compare with ox-LDL treatment group, FGF-21 alleviated cholesterol accumulation in both nc and si-ERK1/2 transfected cells, however, the less of ERK1/2 had no significantly effect on lipid cholesterol accumulation and foam cell formation in THP1 derived macrophages (Fig. 3b and e). To further confirm that ERK1/2 signaling pathway is not involved, we treated THP1 macrophage derived foam cells with PD98059, a phosphorylated ERK1/2 specific inhibitor. With the addition of PD98059, ERK signaling pathway was almost blocked as the decreased phosphorylated ERK1/2 levels (Fig. 3c). But the cholesterol levels remained the same as that in the control group with or without FGF21 treatment (Fig. 3d) These results demonstrate that the functions of FGF21 on THP1 derived foam cells are ERK signaling pathway independent.

### FGF21 suppresses CHOP expression induced by ox-LDL and 7-KC in macrophages

**Fig. 4:**
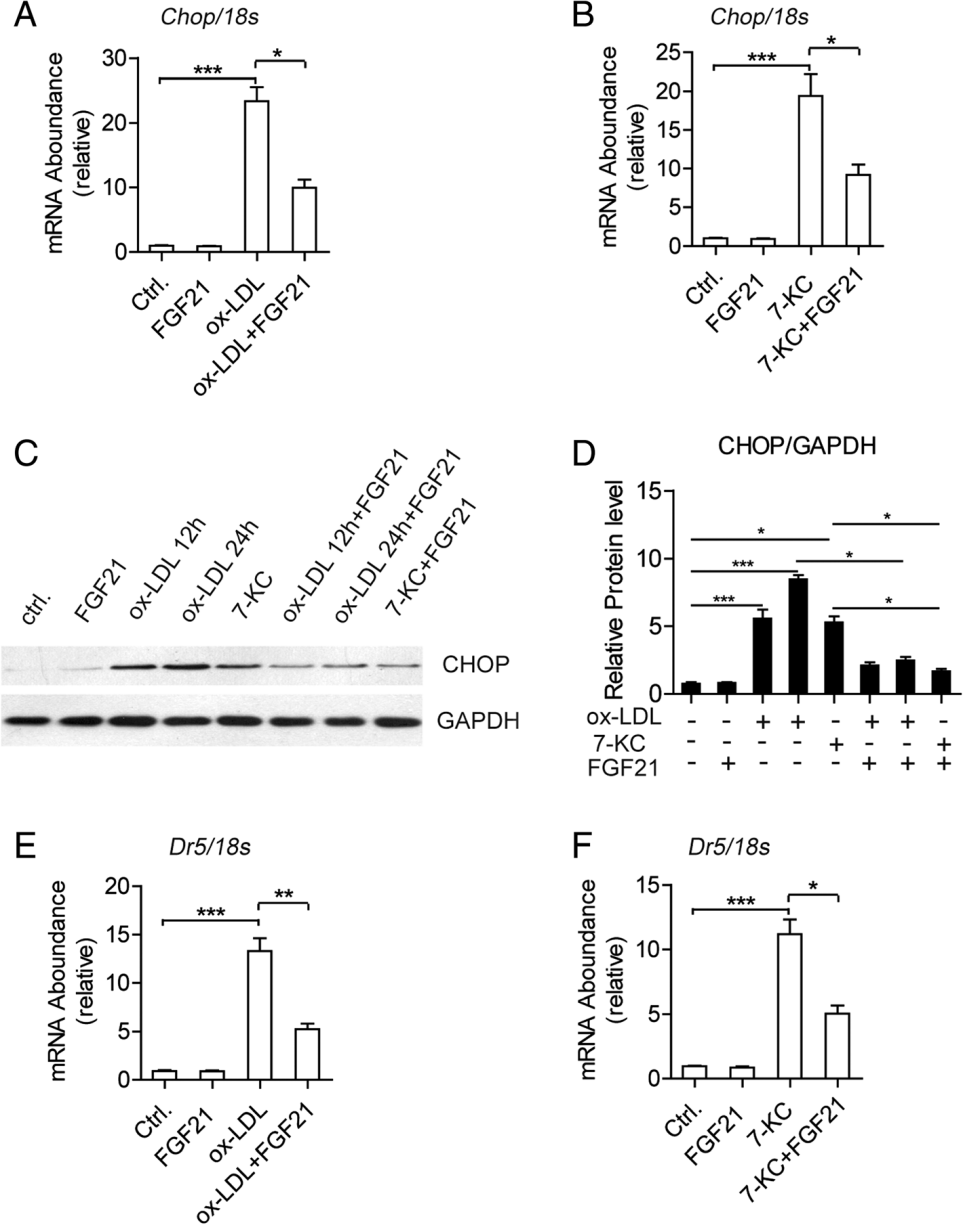
FGF21 suppressed CHOP expression induced by ox-LDL and 7-KC in macrophages. CHOP mRNA level was tested by Real-time PCR in THP1 derived macrophages treated by ox-LDL (**a**) or 7-KC (**b**) in the presence or absence of FGF21 for 24 h, and 18S rRNA was used as internal control. **c** CHOP protein level was tested by western blotting. **d** Quantification of the results in **c**. DR5 mRNA level was measured by Real-time PCR in THP1 derived macrophages treated by ox-LDL (**e**) or 7-KC (**f**) in the presence or absence of FGF21. 18S rRNA was used as internal control. Data are presented as the mean ± S.E. of at least three independent experiments. *, *p <* 0.05, **, *p* < 0.01. N.S, no significance

The apoptosis of macrophages induced by ox-LDL and oxysterols is considered through the activated the Unfolded Protein Response pathway (UPR pathway), and CHOP, the important component in UPR pathway, is considered as the key player. Interestingly, when we tested the RNA and protein levels of CHOP in THP1 derived macrophages, we found that after FGF21 treatment, ox-LDL induced RNA and protein levels of CHOP, were deceased dramatically (Fig. 4a-c). The apoptosis of macrophages induced by 7-KC was also considered to be caused by the upregulation of CHOP. In our data (Fig. 4b-c), FGF21 also suppressed dramatically 7-KC induced CHOP expression. Additionally, Dearth Receptor 5 (DR5), the direct downstream target of CHOP and the main mediator in CHOP-mediated cell apoptosis, decreased significantly after FGF21 treatment in THP1 derived macrophages (Fig. 4c-d).

**Fig. 5:**
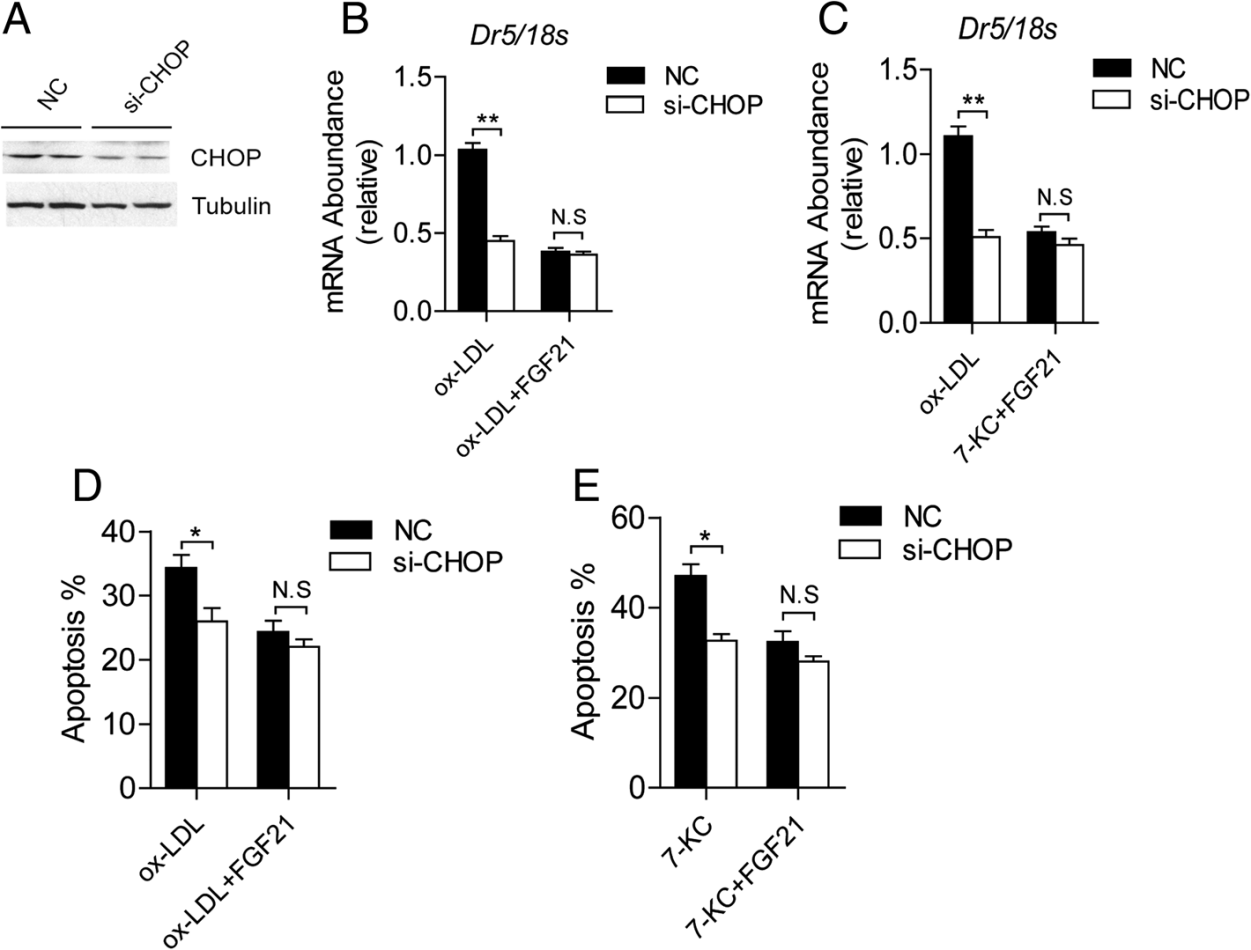
The protection effects of FGF21 on apoptosis were through suppress CHOP signaling in macrophages. **a** The knockdown efficiency of CHOP was tested by western blotting in the THP1 derived macrophages transfected by siRNA. DR5 mRNA level was measured by Real-time PCR in THP1 derived macrophages with si-CHOP transfection treated by ox-LDL (**b**) or 7-KC (C) in the presence or absence of FGF21. 18S rRNA was used as internal control. **d**-**e** Quantification of apoptosis cells in FACS assay of THP1 cells treated as indicated. Data are presented as the mean ± S.E. of at least three independent experiments. *, *p <* 0.05, **, *p* < 0.01. N.S, no significance

To investigate whether the reduction of CHOP is the major reason of FGF21 protection effects, we introduced siRNA specifically targeted CHOP to knockdown CHOP in THP1 derived macrophages. After confirmation of decreased CHOP levels as shown in Fig. 5a, the expression level of DR5 was downregulated in both oxLDL and 7-KC treated macrophages (Fig. 5b-c). With the inhibition on CHOP signaling, the apoptosis of macrophages induced by both oxLDL and 7-KC were dramatically decreased after si-CHOP transfection (Fig. 5d-e). However, the protection of FGF21 on macrophage apoptosis was almost abolished in macrophages when CHOP was knockdown (Fig. 5d-e).

So, the conclusion is that the protection effect of FGF21 on macrophage apoptosis is through CHOP pathway suppression.

## Discussion and conclusion

FGF21, as a member of hormone-like subgroup within the FGF superfamily, is emerging as a key regulator of energy homeostasis and a potential target for the treatment of diabetes, cardiovascular disease, and obesity [4, 8, 23]. A previous study showed that incubation of rodent cardiac microvascular endothelial cells (CMECs) with oxidized LDL led to increased FGF21 expression and inhibited CMEC apoptosis [24]. These results supported the hypothesis that FGF21 function as an endogenous protective factor in the cardiovascular system can improve endothelial function during early stages of atherosclerosis. Moreover, elevated serum FGF21 levels have recently been reported in patients with CHD and are associated with the presence of carotid artery plaques [5, 8]. However, the role of FGF21 in atherosclerosis plaques remains unclear. In this study, we present that FGF21 can protect macrophage against foam cell formation and apoptosis.

In summary, FGF21 repressed the cholesterol accumulation and foam cell formation in THP1 derived macrophage induced by ox-LDL or 7-KC. And the apoptosis of foam cell was decreased with the treatment of FGF21. Moreover,the effect of FGF21 on THP1 derived macrophages was ERK1/2 MAPK pathway independent,and was mediated through suppressing the expression of CHOP and its downstream target DR5. These findings provide evidence for the role of FGF21 in arteriosclerosis and present the new direction for atherosclerosis prevention.
